# Distinctions between sex and time in patterns of DNA methylation across puberty

**DOI:** 10.1186/s12864-020-06789-3

**Published:** 2020-06-03

**Authors:** Sarah Rose Moore, Kathryn Leigh Humphreys, Natalie Lisanne Colich, Elena Goetz Davis, David Tse Shen Lin, Julia Lynn MacIsaac, Michael Steffen Kobor, Ian Henry Gotlib

**Affiliations:** 1 Department of Medical Genetics, University of British Columbia | BC Children’s Hospital Research Institute, 938 W 28th Ave, Vancouver, BC V5Z 4H4 Canada; 2 Department of Psychology and Human Development, Vanderbilt University, 230 Appleton Pl, Nashville, TN 37203 USA; 3 Department of Psychology, University of Washington Seattle, Guthrie Hall (GTH), 119A 98195-1525, Seattle, WA 98105 USA; 4 Department of Psychology, Stanford University, 450 Jane Stanford Way, Stanford, CA 94305 USA

**Keywords:** DNA methylation, Puberty, Epigenetic regulation, Testosterone, Gonadal hormones, Sexual differentiation

## Abstract

**Background:**

There are significant sex differences in human physiology and disease; the genomic sources of these differences, however, are not well understood. During puberty, a drastic neuroendocrine shift signals physical changes resulting in robust sex differences in human physiology. Here, we explore how shifting patterns of DNA methylation may inform these pathways of biological plasticity during the pubertal transition. In this study we analyzed DNA methylation (DNAm) in saliva at two time points across the pubertal transition within the same individuals. Our purpose was to compare two domains of DNAm patterns that may inform processes of sexual differentiation 1) sex related sites, which demonstrated differences between males from females and 2) time related sites in which DNAm shifted significantly between timepoints. We further explored the correlated network structure sex and time related DNAm networks and linked these patterns to pubertal stage, assays of salivary testosterone, a reliable diagnostic of free, unbound hormone that is available to act on target tissues, and overlap with androgen response elements.

**Results:**

Sites that differed by biological sex were largely independent of sites that underwent change across puberty. Time-related DNAm sites, but not sex-related sites, formed correlated networks that were associated with pubertal stage. Both time and sex DNAm networks reflected salivary testosterone levels that were enriched for androgen response elements, with sex-related DNAm networks being informative of testosterone levels above and beyond biological sex later in the pubertal transition.

**Conclusions:**

These results inform our understanding of the distinction between sex- and time-related differences in DNAm during the critical period of puberty and highlight a novel linkage between correlated patterns of sex-related DNAm and levels of salivary testosterone.

## Background

Sexual differentiation is largely understood to be induced by adrenal and gonadal hormones operating early in the postnatal period and again during puberty. Indeed, puberty is characterized by a drastic neuroendocrine shift signaling physical changes, such as the development of secondary sex characteristics and the redistribution of adipose tissue, and organizational/activational effects of adrenal and gonadal hormones on brain development [1, 2]. With these changes, there are also robust sex differences in human physiology and in the prevalence and symptomatology of a number of mental and physical health disorders [3]. How these drastic shifts in biology are encoded within the genome and are developmentally regulated, however, is not well understood.

At the level of the genome, males and females are indistinguishable outside of the sex chromosomes. In contrast, across the autosomes, it appears that there are sex-specific patterns of genetic regulation. Specifically, the transcriptome [4–6] and epigenome [7–9] are characterized by robust sex differences outside of the sex chromosomes. Indeed, sex differences in surges of pubertal hormones lead to sexual differentiation via regulatory pathways, affecting the transcriptome and epigenome in a sex-specific manner [10, 11]. The primary androgen released from the gonads, testosterone, initiates genital development in males, but rises substantially and plays a role in developmental changes in both males and females [12]. Testosterone crosses the blood brain barrier to drive sexual differentiation of brain structure and organization [13], and studies in humans have shown associations among testosterone levels in puberty and structure and function of both cortical and subcortical regions of the brain [14–16]. Moreover, testosterone activates the androgen receptor, a transcription factor protein that, upon binding, translocates to the nucleus to stimulate transcription of a host of androgen responsive genes. Although pubertal levels of gonadal hormones have been studied extensively in relation to physical and neural development in adolescents [17], the functional genetic pathways that are regulated to produce these changes are just beginning to be explored in humans [18–22].

DNA methylation (DNAm) is an epigenetic mark that is highly intertwined with biological development. DNAm refers to the addition of a methyl group to a cytosine base commonly adjacent to a guanine base (i.e., a CpG dinucleotide). Beginning in fertilization, the genome undergoes global demethylation followed by epigenetic reprogramming, in which the cells and tissues of the developing embryo differentiate according to distinct patterns of DNAm [23]. Although the patterns of DNAm that arise at cellular differentiation remain stable and are reproduced in daughter cells, changes to the methylome continue to accumulate across the lifespan [24, 25]. For significant developmental reorganizations such as sexual differentiation, dynamic shifts in DNAm may be particularly informative of the genetic pathways driving this biological plasticity.

In this investigation we focused on a gene network analysis of DNAm linked to sexual differentiation during the pubertal transition when boys and girls diverge in phenotypes at both physiological and behavioral levels. Because gene regulation is organized hierarchically [26], network approaches are able to identify biological pathways, or ‘modules’ composed of many units, or ‘nodes,’ that shift together in relation to a developmental period or disease state [27]. Thus, for example, when one gene’s protein product regulates the expression of another set of genes, the cascade of effects and the interrelations among many downstream regulatory effects can be modeled together, and the driving ‘hub’ genes, sitting at the top of the network hierarchy, can be identified. In the first step, differential methylation analysis is conducted to detect individual nodes, which are then carried forward to the second step: a network analysis to identify the connections or correlational structure among the nodes, as well as the most strongly interconnected hubs.

We quantified DNAm in saliva at two time points across the pubertal transition within the same individuals to examine differential methylation followed by network analysis. We further linked network modules to assays of salivary testosterone, a reliable diagnostic of free, unbound hormone that is available to act on target tissues [12, 28, 29]. Previous studies have explored and identified DNAm sites that change across the pubertal transition, which are generally common between males and females [3, 18]. However, it is unclear whether DNAm sites that are different between males and females, in particular, are relevant to shifting biological states in the sexes at puberty, such as pubertal stage and hormonal levels.

To explore how sex differences fit into the shifting regulatory landscape at the critical transition through puberty, our strategy targeted two domains of differential methylation for network analysis that may drive the processes of sexual differentiation: 1) sex-related sites, which demonstrated differences between males and females at Time 1 (T1) or Time 2 (T2); and 2) time-related sites in which DNAm shifted significantly from T1 to T2. Because previous studies have focused solely on sites that change over puberty, and have not directly examined sex differences, we aimed to contrast and characterize sites in each domain in order to gain a more comprehensive picture of genetic regulation of pubertal shifts in boys and girls. We explored the independence of time- and sex-related DNAm sites, the correlated networks of time- and sex-related DNAm sites and the specific patterns that drove these networks, and their associations with pubertal maturation, as assessed by pubertal stage, salivary testosterone, and overlap with androgen response elements.

We found that sex-related sites were largely independent of time-shifting sites and, further, formed correlated networks of DNAm patterns that synchronized with salivary testosterone levels later in the pubertal transition. Time-related sites were associated with testosterone levels in males, and were correlated with pubertal stage. These results inform our understanding of the distinction between sex- and time-related differences in DNAm at this period and highlight a novel linkage between sex-related co-methylated gene networks and circulating testosterone in saliva.

## Results

Participants were selected based on pubertal stage (i.e., self-reported Tanner staging), matching males (*n* = 47) and females (*n* = 71) on Tanner stage at T1 using the average Tanner scores for pubic hair and breast/testes development. Participants returned for the second time point (T2) an average of 1.97 years later (sd = 0.33, range 1.29–3.37 years; Supplementary Fig. 1A). Participants provided saliva samples for DNAm quantification via the Illumina EPIC array and additional samples for assay of salivary testosterone (males and females; Supplementary Fig. 1B). We used the following strategy in analyzing our data: 1) conduct a differential methylation analysis to separately identify sites that were associated significantly with sex (at T1 and T2) and time (i.e., that shifted between time points); 2) assess the independence or overlap between sex- and time-related sites, and characterize effect sizes, direction, and genomic context; 3) carry forward significant sex- and time- sites to explore co-methylated gene networks, summarized by network ‘modules’ and driving network hubs; 4) further probe co-methylated network modules and hub CpG sites for biological significance by testing for associations with Tanner stage and salivary testosterone, and enrichment for androgen response elements.

### Step 1: differential methylation analysis to identify sex- and time-related DNAm sites

**Fig. 1:**
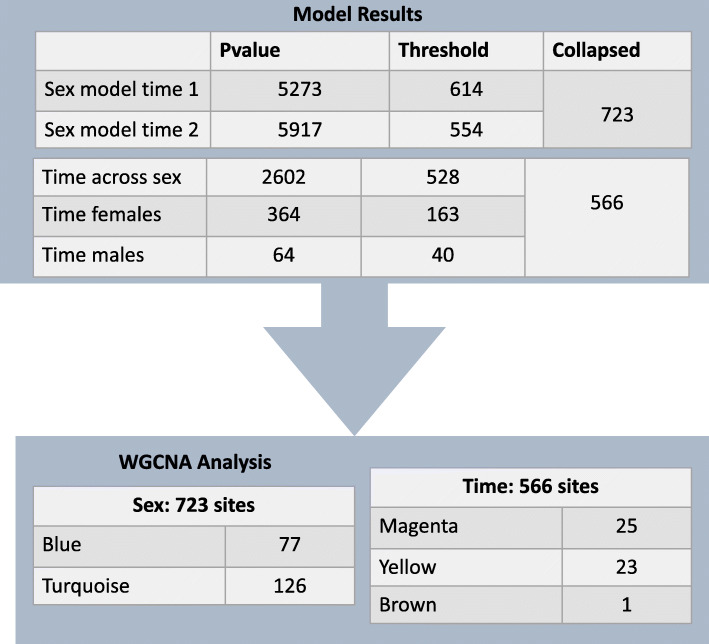
DNA methylation sites identified by statistical models and carried forward to network analysis. WGCNA = weighted correlational network analysis

First, to identify individual DNAm sites that differ between males and females, we conducted statistical models regressing DNAm at each site (794,811 sites) onto sex, controlling for age, ethnicity, and cell type proportions (bioinformatically computed using Hierarchical EpiDISH; see methods) separately for each time point. At the first time point, 5273 DNAm sites were significantly related to sex (adjusted *p* < 0.05); at the second time point, 5917 sites were significantly related to sex (adjusted p < 0.05) after multiple test correction (for all multiple tests we used the false discovery rate Benjamini-Hochberg procedure; Fig. 1). Across both time points, there were 3174 overlapping sex-related sites. This significant overlap may suggest sex-related sites are common across stages of puberty.

Next, we conducted models to identify the sites that were shifting within individuals over the pubertal transition using a mixed model, consistent with prior pubertal DNAm studies that assessed DNAm at early and late pubertal stages (i.e. [18, 30],). We conducted mixed models to calculate the effect of time point across samples controlling for the interval (in years) between T1 and T2, ethnicity, and cell type proportions, with individual subject ID added as a random effect. In addition, we compared these results to the effect of time in separate models conducted for males and females and followed up on significant sites to assess what variables corresponded to changes in DNAm at time-related sites (see methods). In a full model testing for the effect of time across males and females and controlling for covariates, time had a significant effect for 2602 probes after multiple test correction. In females, 364 sites shifted significantly with time (91.45% overlap with the full model) and, in males, 64 sites shifted significantly (90.63% overlap with the full model; Fig. 1). Models split by sex are substantially less powered and, given the large degree of overlap in sites, we collapsed significant sites across the full, male, and female time models for network analyses (see below). We conducted follow-up models on these sites in order to assess what variables corresponded with shifting DNAm patterns: we found that sex predicted shifts only at one site, whereas the age intercval between time points predicted shifts at 20 sites. Smoking and changes in Tanner Stage did not predict DNAm change. Thus, overall, time sites that were moved forward for network analysis for comparison with sex-related sites were mostly independent of sex and age, and entirely independent of pubertal stage (see Methods). All significant model results are presented in Supplementary Table 5. In our next set of analyses, we compared these time- and sex-related sites to assess their independence and characterize effect sizes, directions, and genomic location in the context of puberty.

### Step 2: assess independence of sex- and time-related DNAm sites and characterize effect size, direction, and genomic context

**Fig. 2:**
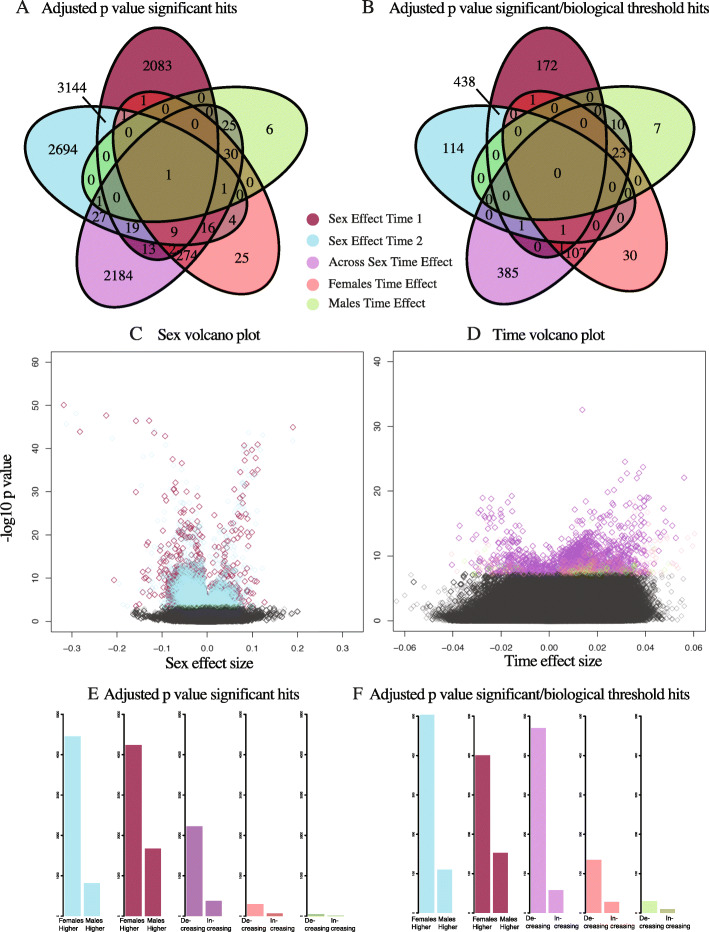
Overview of significant and biologically thresholded hits across sex and time models. A) Venn diagram showing overlap of significant results (adjusted *p* value< 0.05) from models testing the effect of sex at T1, sex at T2, time across sexes, time in females, and time in males. B) Venn diagram showing how overlapping results shift when applying a biological threshold of delta beta > 0.05 for sex, and > 0.02 for time (i.e., reducing the model results to those meeting both significance and effect size thresholds). C) Volcano plot for sex models at T1 and T2, with effect size on the x axis and -log10 of the *p* value on the y axis. Significant sites with larger effects are visisble in the top left and right quadrants. D) Volcano plot for time models across sex, only females, and only males. *Note.* Different x axis scale for Fig. 2C and D. E) Count of adjusted *p* value significant probes found across models for sites that are higher in females versus higher in males. F) Count of adjusted p value significant probes meeting biological thresholds across models that are increasing or decreasing with time. *Note*. Different y axis scale for Fig. 2E and F

Co-methylation network analysis is driven by patterns of connections among the nodes identified at the differential methylation analysis stage described above. Thus, to gain a better understanding of the patterns of DNAm in sites related to sex and to time, we assessed the overlap of sex- and time-related sites, the size and direction of effects, and their location in relation to genes (i.e., genomic context). To assess the independence of sex- and time-related sites and effect sizes, we assessed overlap both for multiple test correction significant hits (Fig. 2A) and for sites that surpassed a biological threshold (Fig. 2B). Overall, the majority of significant sites are specific to sex or time; only 46 probes overlapped between sex and time models (1% of unique sex sites and 2% of unique time sites). To further probe effect size, we applied a standard biological threshold of an absolute delta beta greater than 0.05 [31]. A total of 723 unique sex-related sites (14%) exceeded this threshold. In contrast, only four sites (0.2%) survived a delta beta > 0.05 threshold in the time models (none of which overlapped with sex-related sites), demonstrating that, overall, time-related effect sizes are smaller than are sex-related effect sizes. When applying a biological threshold to time effect models, more sites were found for female and male specific time effect models relative to the *p* value threshold, suggesting that some sites from across sex time effect models were larger in either males or females. Fig. 2C and D show the effect sizes from different models relative to significance: the signal of sex highly surpasses that of time shifts. This is consistent with the epigenetic aging literature, in which average shifts in DNAm of aging-related sites are reported to be 3.2% across a span of 20 years [32]. Due to the overall smaller effects of time, we set the biological threshold for time-shifting probes to an absolute delta beta greater than 0.02 for further analysis, which increased the time-related probes to 566 (19% of significant sites). With respect to overlap of significant sites with larger effects, sex-specific sites were largely independent of sites that shifted significantly from T1 to T2: in fact, there were only three CpGs that showed differential methylation between both sex and time points that met their respective biological thresholds. Overall, these comparisons indicate that the effects of sex are largely distinct from the effects of time on DNAm in saliva during this phase of puberty.

Next, we examined sex- and time-related sites for trends in effect direction and genomic context of CpG sites. The trends in direction of sex-related sites were largely similar at T1 and T2 (Fig. 2E and F): more sites had higher DNAm in females than in males. Similarly, across both sexes and within females and males separately, time shifts were largely due to sites that decreased from T1 to T2. All significant effects of sex at T1 and T2, and significant effects of time point, were dispersed across the autosomes (i.e., significant DNAm sites were identified across chromosome 1–22).

To further characterize the genomic context of sex- and time-related effects, we collapsed significant sites that met the respective biological threshold (sex 0.05, time 0.02) identified across sex models (T1 and T2: 723 sites) and time models (all, females, and males: 566 sites) to compare to the full background of tested sites on the EPIC array for enrichment for genomic locations (i.e., promoter, gene body, intergenic, etc.) as well as mQTLs (see Methods). Sex-related probes were enriched in gene bodies (adjusted *p* value = 2.73e^− 06^), intergenic spaces (adjusted p value = 4.01e^− 05^), and transcription start sites (within 1500 bp; adjusted p value = 0.04), and time-related probes were enriched for gene bodies (adjusted p value = 0.01) and transcription start sites (within 200 bp; adjusted p value = 0.01; Supplementary Table 2). Both sex- and time-related sites were enriched for mQTLs identified in buccal cells (see Methods). Next, we were interested in the distinct network structures that might correspond to these separate sets of sites and whether and how they reflect processes of sexual differentiation during puberty.

### Step 3: network analysis to identify co-methylated gene networks

Sex- (723) and time- (566) related sites meeting respective biological thresholds were next carried forward to explore correlated network structure. We applied weighted correlation network analysis (WGCNA) to identify possible co-methylated gene networks from sex- and time-related sites. WGCNA assesses patterns of correlated DNAm levels across many probes by estimating gene clusters, or ‘modules’ summarized by a first principal component or eigengene. These modules represent a correlated network of DNAm sites in terms of their interrelations across samples. In two WGCNA analyses, we tested DNAm sites that met the above set biological thresholds (723 sites > 0.05 for sex and 566 sites > 0.02 for time). We repeated analyses for all adjusted *p* value significant probes to confirm the robustness of network results (5273 sites for sex and 2639 sites for time).

#### Sex network analysis

**Fig. 3:**
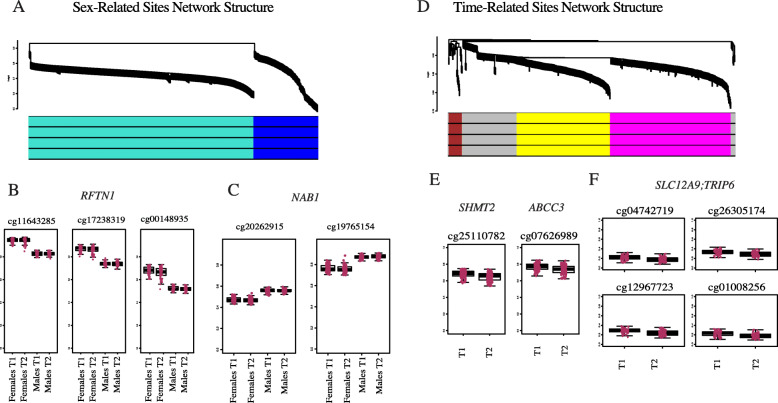
Network structure and example hub CpG patterns from sex- and time-related CpG sites. A) Network structure of sex-related probes identified by WGCNA. The hierarchical cluster tree shows co-methylation modules, with each leaf in the tree representing an individual CpG. B) Example boxplots of beta values of three hub CpGs from the turquoise module annotated to *RFTN1*. C) Example boxplots of beta values of two hub CpGs from the blue module annotated to *NAB1*. D) Network structure of time-related probes identified by WGCNA. Note gray color means no cluster identified. E) Example boxplots of beta values of two hub CpGs from the blue module annotated to *SHMT2* and *ABCC3*. F) Example boxplots of beta values of four hub CpGs from the turquoise module annotated to *SLC12A9;TRIP6*

We first conducted WGCNA on all sex-related probes with absolute delta betas > 0.05 (723) across both time points on beta values that were corrected for cell type proportions. This identified two modules, the ‘blue’ and the ‘turquoise’ modules, representing co-methylated gene networks (Fig. 3A), summarized in the following by module eigengenes, the first principal component of which captures how modules relate to one another and across individuals. We explored the top ten ‘hub’ CpGs sites (i.e., those with the highest eigen-based connectivity) between the two modules. From the turquoise module three CpGs were identified as hubs within the gene body of *RFTN1* (Raftlin, Lipid Raft Linker 1) (Fig. 3B). This is a Protein Coding gene related to double-stranded RNA binding. All three CpG sites had higher DNAm levels in females than in males. In the blue module, two CpGs were identified as hubs from the gene body of *NAB1* (NGFI-A Binding Protein 1), a protein coding gene involved in transcription factor binding and implicated in Breast Cancer. These CpGs were more highly methylated in males than in females (Fig. 3C). Supplementary Table 3 shows the patterns of all high-connectivity probes, with kMeans < 0.7 from WGCNA analyses. This table shows that there are also highly connected probes that show opposite trends from the top hub CpG examples in each module. Repeating the analysis with the full set of adjusted *p* value significant probes confirmed the same correlational structure and common hub CpGs for both modules. Each module can be summarized by a principal component (PC), and then the similarly of modules between different network analyses can be assessed by correlating these top PCs from each module. The correlations between top module PCs produced by the *p* value significant sites in a network analysis versus module PCs produced by a network analysis on the reduced set of biological thresholded sites is shown in Supplementary Fig. 2A.

A functional pathway analysis of top kME genes from each module (77 sites in blue; sites 126 in turquoise) indicated that sites composing the blue module were enriched for mitotic and cell cycle functions, and sites composing the turquoise module were enriched for protein transport and localization to membrane raft and T cell antigen processing (Supplementary Table 4).

#### Time network analysis

We conducted WGCNA on all time-related CpGs that met the biological threshold from the general, female, and male models (> 0.02, 566); this yielded three modules (Fig. 3D). Although we combined sites from the male and female models with the general model, none of the three modules yielded significant sex differences (Brown, t = 0.83, *p* = 0.41; Yellow, t = 0.22, *p* = 0.83; Magenta, t = − 0.55, *p* = 0.58), suggesting that the major variability in DNAm shifts across time are consistent between males and females. The brown module had only one DNAm site that met a kME threshold >.7, but all top-10 hub CpGs increased over time. Three DNAm sites were associated with the meiotic recombination protein *REC8* from the kleisin family of structural maintenance of chromosome protein partners; however, such processes would only take effect in germ cells. Of the yellow module driving hub CpG sites, seven are located within seven different genes and three are intergenic. Example DNAm patterns at T1 and T2 are plotted in Fig. 3E: a site within *SHMT2*, a gene encoding a mitochondrial enzyme responsible for glycine synthesis, and a site associated with *ABCC3*, which encodes an ATP-binding transporter. All of the most highly interconnected DNAm sites within the yellow module decreased in DNAm from T1 to T2. In the magenta module (examples plotted in Fig. 3F), five different highly connected CpGs located in the *SLC12A9-TRIP6* gene region were identified as hubs, consistent with a previous report [19]. *SLC12A9* (Solute Carrier Family 12 Member 9) is a protein coding gene implicated in cation and chloride symporter activity, and *TRIP6* (Thyroid Hormone Receptor Interactor 6) encodes a protein recruited to the plasma membrane to regulate lysophosphatidic acid-induced cell migration. All show decreasing DNAm patterns from T1 to T2. Supplementary Table 3 shows the patterns of all high-connectivity probes, with kMeans < 0.7 from the time modules. All highly connected CpGs from the yellow and magenta modules decreased in DNAm over time, and the single CpG from the brown module increased over time.

Repeating the analysis with the full set of adjusted *p* value significant probes confirmed the same correlational structure and hub CpGs for the yellow and magenta modules; however, the brown module was not well represented in the second set of probes (Supplementary Fig. 2B). In combination with the low number of probes (1 site) that met a kME interconnectivity cut-off, these results suggest that the brown module is not as robust in its correlational structure. Moreover, the most highly connected DNAm sites of the brown module fall within a gene in which the protein is only necessary in germ cells, which is inconsistent with the adolescent stage; thus, we focused follow up investigations on the yellow and magenta modules.

Functional pathway analysis of top kME genes from each robust module (25 sites in yellow; 23 sites in magenta; 1 site in brown) showed that the yellow module was enriched for interleukin-1 receptor complex, the magenta module was enriched for glycine biosynthetic process, and glycine hydroxymethyltransferase activity, and the brown module for protein localization to M-band (Supplementary Table 4).

Now with correlated network modules to move forward, our next set of analyses explored the functional links of sex- and time-related sites to additional measures of pubertal development and hormones.

### Step 4: explore functional links of sex- and time-related co-methylation network modules

To explore the biological relevance of sex and time co-methylated network modules, we explored the associations between 1) sex- and time-related modules; and 2) pubertal stage and salivary testosterone. Following up on links with testosterone levels, we further examined enrichment of sex- and time-related sites for androgen response elements.

#### Module correlations with pubertal stage

**Fig. 4:**
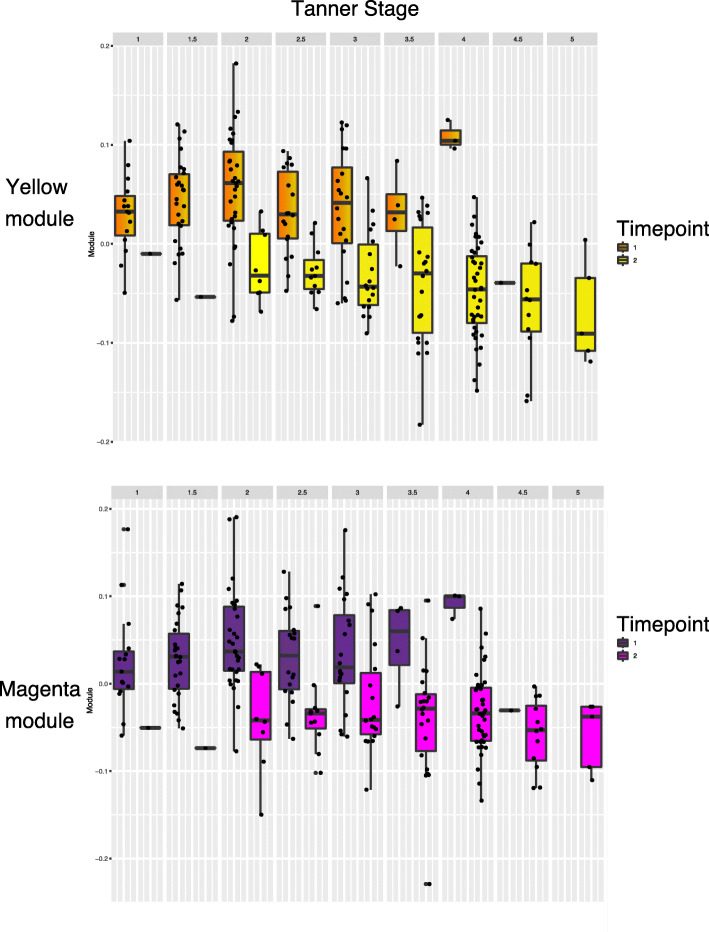
Boxplots displaying time-network module scores across Tanner stages for T1 and T2. Module scores decrease over time, but show nonsignificant, opposite trends for each time point (increasing at T1 and decreasing at T2)

We correlated module eigengenes from the sex- and time-correlated networks to assess if and which sets of correlated CpG sites were informative of pubertal stage. For the sex-correlated network at T1, the turquoise module was significantly correlated with Tanner stage (r = 0.19, *p* = 0.03); the blue module was not correlated with Tanner stage (r = − 0.17, *p* = 0.06). Neither of the sex modules was significantly related to Tanner stage at T2 (blue module, r = − 0.11, *p* = 0.29; turquoise module, r = 0.12, *p* = 0.27). In contrast, both time-related network modules were strongly correlated with Tanner stage (yellow module, r = − 0.50, *p* = 5.49e-16; magenta module, r = − 0.37, *p* = 4.38e-09). Fig. 4 presents Tanner stage for each time point plotted against time module scores. Interestingly, although there is an overall negative association of each network module with Tanner stage, the network structure accounts for Tanner staging differently at each time point, such that there is an increasing association at T1 and a decreasing association at T2, these trends, however, are not statistically significant (Supplementary Table 5).

#### Module correlations with testosterone

**Fig. 5:**
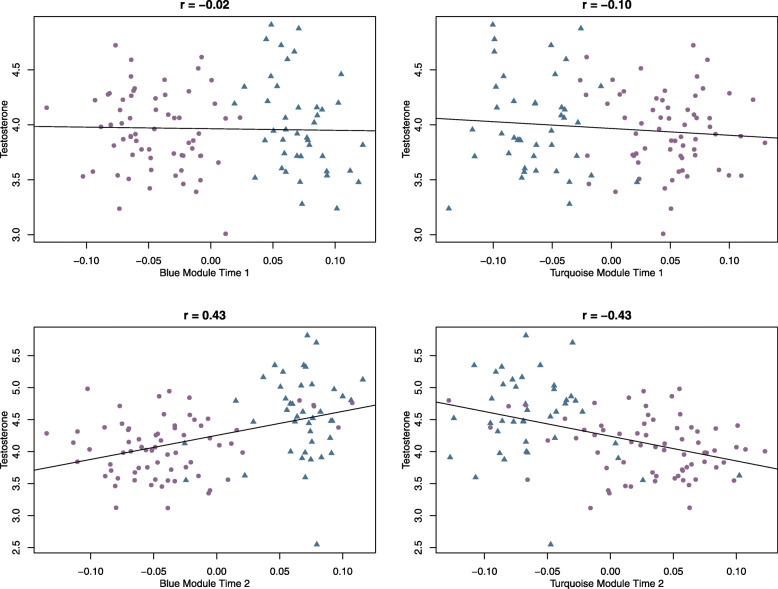
Relation between salivary testosterone and sex module scores. Correlations between 1) blue and turquoise modules estimated by WGCNA on sex-related probes and 2) testosterone at T1 and T2. Module scores show clear distinctions between males and females at T1 and do not correlate with testosterone. At T2 these scores no longer cleanly distinguish males and females and correlate significantly with testosterone

Testosterone is a driving hormone in sexual differentiation over the pubertal transition. To explore potential links of sex- and time-related network modules, we correlated network eigengene module scores with testosterone levels at each time point in a subsample of participants with salivary testosterone data (*n* = 106 at T1 and *n* = 111 at T2). These correlations are presented in Fig. 5. Neither module from the sex-related DNAm network was significantly correlated with testosterone at T1, but was significantly correlated at T2. Moreover, whereas males and females demonstrate highly distinct module membership at T1, they are less distinguishable at T2, as modules shift into tight correlations with testosterone, especially for the turquoise module. This suggests that the correlated networks represented by the modules capture a shift in DNAm levels from T1 to T2 that defines shifting biological status in terms of circulating available testosterone beyond biological sex.

Given these tight correlations of modules with testosterone, we next assessed whether our sex-related CpGs that reached a biological threshold of > 0.05 (723 probes) were individually related to testosterone at T2 but not at T1 after correction for multiple comparisons (Supplementary Table 1). After covarying for the effects of age, ethnicity, and cell type proportions (but not for sex, which is colinear with hormone levels), no probes were significantly related to testosterone at T1. At T2, 334 probes (46%) were significantly predicted by testosterone. All ten top hub CpGs from both modules and 183/203 of the probes from the modules with a kMeans < 0.7 were overlapping with probes that were significantly related to testosterone. Correlations between the top 10 hub CpGs from both modules and testosterone at T1 and T2 are presented in Supplementary Fig. 3.

**Fig. 6:**
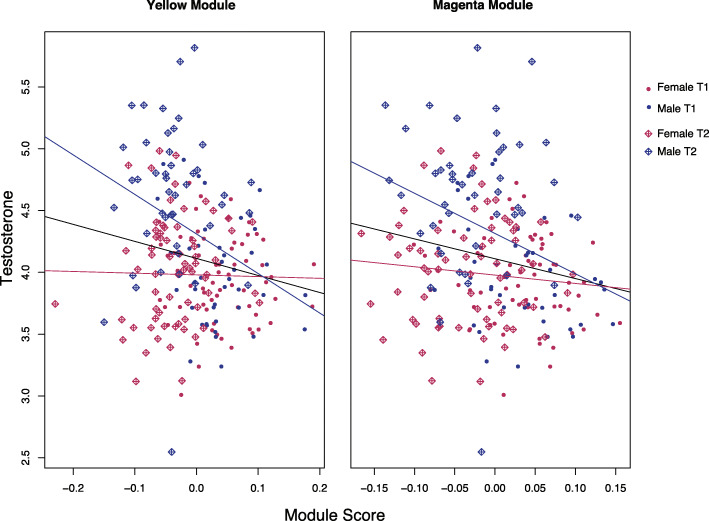
Relation between salivary testosterone and time module scores. Points distinguished by time point, with greater variability in module scores at T1 relative to T2. With increases in each module score, testosterone levels increase. The relation is stronger for males than for females, shown by separate regression lines for males and females

Next, we assessed correlations between time modules and testosterone. The yellow (r = .18, *p* = 0.009) and magenta (r = − 0.20, *p* = 0.003) modules both were significantly correlated with testosterone levels. Variability in module scores was much greater at T2 relative to T1 (Fig. 6), with T1 male and female module scores clustering close together for both the yellow and magenta modules. Module scores are more spread apart and differentiate males and females at T2 in both modules. Similar to sex-related CpGs above, we tested whether DNAm levels at individual time-related CpG sites significantly correlated with testosterone at time 2 but not at time 1. No individual CpG sites were significantly related to testosterone levels at time 1, and only one CpG site out of 566 significantly related to testosterone at time 2 (Supplementary Table 1). Thus, in contrast to sex-related CpG sites, the associations of time-related sites with testosterone levels are not as strong.

Separate regression lines for males and females show that module score associations with testosterone levels are stronger in males, supported for both the yellow and magenta modules (Fig. 6). Regressing testosterone levels on the interaction between module score and sex was statistically significant for both modules (Supplementary Table 5). Thus, although time network modules were largely independent of sex, these correlated networks of DNAm captured variability in testosterone levels to a greater extent for males.

Finally, to assess if there was evidence for epigenetic regulation of gene networks due to testosterone effects, we tested for enrichment of sex- and time-related sites for androgen response elements (AREs) using data from Remap (see methods). 85% of sex-related sites and 87% of time-related sites were found within androgen response elements, both of which were greater than expected by chance (sex-related sites: χ^2^ = 238.57, *p* < 2.2e^− 16^; time-related sites: χ^2^ = 232.77; p < 2.2e^− 16^) by comparing to a baseline of the average overlap for 10 random draws of sites from quality controlled probes. Taken together, it appears that DNAm networks of sexually differentiating sites and time-shifting sites at the pubertal junction are both linked to fluctuating testosterone levels and are found within androgen response elements.

## Discussion

Puberty sets into motion dramatic sexual differentiation between human females and males in terms of physiology, as well as sex differences in the onset, symptomology and prevalence rates of disease and disorder [3, 33, 34]. In this study we examined DNAm as a marker of regulatory changes in sexual differentiation during the pubertal transition. We showed that sex-specific patterns of DNAm, captured as co-methylated network ‘modules’, shift from early to later puberty, falling into tight correlation with salivary testosterone, an indicator of available, unbound hormone for action across tissues. In contrast, network modules composed of DNAm sites that fluctuated across time at the pubertal junction were more strongly linked to pubertal developmental stage. These results add to a growing literature demonstrating that DNAm is intertwined with biological development and may inform genetic loci to be targeted in future studies in order to advance our understanding of how biological differences between males and females are initiated at puberty.

Whereas prior research on DNAm across puberty has primarily focused on individual sites in relation to pubertal timing or combined sets of sites as predictors of pubertal timing [18, 19], we applied network analysis to two time points of DNAm to identify nuanced shifting in DNAm patterns over this critical transition. We collapsed all sexually differentiated sites across time points, and all time-shifting sites across sexes, to explore correlated patterns of DNAm variability. DNAm sites formed cohesive networks, with correlated patterns of sex-related sites falling into tight correlation with salivary testosterone later in puberty when levels of fluctuating hormone increase in both sexes. Individuals clearly cluster within these module scores by biological sex at T1, but shift at T2 in association with testosterone, such that boys and girls are no longer in distinct groups. In contrast, correlated patterns of time-shifting sites captured variability in pubertal stage despite no associations of individual sites with pubertal stage. Time-shifting sites further demonstrated mild associations with testosterone that were driven by boys. These findings demonstrate the distinction between sex- and time-specific patterns in DNAm in terms of the biological changes of sexual differentiation and pubertal development, and suggest that sex-related sites should also be considered in the study of genetic regulation of pubertal processes.

Notably, these results depend on subtle changes in the structure of interrelations among sexually differentiated and time-shifting CpG sites, highlighting the benefit of using machine-learning approaches to identify correlated networks of DNAm. WGCNA yielded modules that define how individual variability among sex- and time-related probes corresponds to individual variability in testosterone, a critical biological driver of sexual differentiation. In previous, well-powered work (over 400 individuals of each sex) by Arathimos and colleagues that aimed to identify testosterone-related DNAm sites in blood in adolescents, the signal of testosterone was not strong enough to identify any individual sites after correction for multiple tests [35]. In contrast, another study identified a DNAm signature of 999 CpG sites in blood in association with testosterone levels, but only in boys, which may be attributable to targeting the heart of pubertal changes in earlier adolescence than did Arathimos and colleagues [35]. In the current study, the minor shifts in the correlational structure of individual sex-related CpGs from T1 to T2 that relate to testosterone were not captured as time-shifting sites, and our post-hoc tests for associations of sex-related sites with testosterone would not have withstood genome-wide multiple test correction of over 700,000 CpG sites. Moreover, the sites identified in relation to sex and time were both highly enriched for androgen response elements, suggesting these shifting correlational structures across puberty may be driven by the regulatory effects of testosterone via activation of the androgen receptor regulatory cascade. Thus, we further show that subtle, small effects in DNAm data may be biologically meaningful [36].

Beyond the differences in the relations with testosterone levels and pubertal stages, the distinctions between sex-related and time-related DNAm sites were apparent at every stage of analysis. First, the sets of sites that met statistical significance and biological thresholds were independent, with the biological thresholds highlighting the much larger effects of sex relative to time. Second, the correlated DNAm network modules and driving hub CpG sets had different associated biological pathways. Sex-related network modules were enriched for cellular organization, communication, brain morphogenesis, social behavior and intraspecies interaction, whereas time-related sites demonstrated primarily cellular morphogenesis and cell signaling. Third, DNAm sites driving sex-related correlated networks were strongly linked to testosterone levels, whereas time-related sites corresponded to physical maturation as assessed by Tanner stage. Thus, at least in saliva, it appears that the processes of pubertal changes reflected in sex differences versus time-shifts are independent with different functional implications.

In addition to revealing these distinctions between sexual differentiation and time-shifting DNAm patterns, our results confirm a number of previous observations in the DNAm literature of sex differences and pubertal development. First, we replicate previous work that has documented robust sex differences in DNAm across the genome using adult post mortem prefrontal cortex tissue [37], peripheral blood [38], cord blood [39], and saliva [40]. Second, the driving hub CpGs of a time network module were previously reported in linkage to pubertal development by Almstrup and colleagues, who demonstrated that a region of *SLC12A9/TRIP6* shifts across puberty consistently between boys and girls in blood [19]. Similarly, we found that time-shifting sites were correlated with Tanner stage, corroborating prior research showing that DNAm sites can be combined as predictors of pubertal timing [18, 19].

Third, we found a greater proportion of sex-related CpG sites to have higher DNAm in females than in males [37, 39], and more sites that shifted significantly across puberty in females than in males [18]. It is possible that females show greater fluctuations between time points due to variability driven by the ovarian cycle. Indeed, unlike boys, once girls reach menarche, the regulatory endocrine processes of pubertal changes become tied to the ovarian cycle [21]. More longitudinal DNAm work and precise measurement of the ovarian cycle at the time of sample collection is crucial in order to gain a more comprehensive understanding of these developmental differences between males and females.

Although we obtained results consistent with those of previous studies, it is important to note that the ability of DNAm in saliva to inform DNAm patterns in other tissues is still unclear, and that saliva as a surrogate tissue is not a direct index of the neural and developmental processes that are of key importance to pubertal changes [30]. It is possible, however, that common genes are regulated by testosterone across tissues during puberty; thus, DNAm in saliva may capture neurodevelopmental pathways in sexual differentiation by proxy.

Functionally, the interpretation of DNAm in peripheral tissues in human research is difficult. Nevertheless, whether the observed DNAm shifts are causes or byproducts of pubertal developmental processes, the -omics of sex differences may be informative for personalized medicine applications [34, 41]. With the emergence of sexual differentiation, there are sex-related disparities in health, such as diabetes, cardiovascular disease, and mood disorders. Deciphering how epigenetic architecture diverges during this critical window of sexual differentiation may yield insights concerning the origin and genetic regulation of sex-related risks [34].

From a basic research standpoint, the stark differences in the sexes at the level of DNAm across the autosomes, in contrast to indistinguishable sex differences in genetic variability, is puzzling. Why do females demonstrate broadly higher DNAm levels relative to males across tissues? X chromosome inactivation relies on hypermethylation, and it is possible that this originating mechanism also affects the rest of the genome, becoming engrained across cells during embryogenesis. Thus, the overall discrepancy would be maintained throughout development, and subtle shifts in sexually differentiated sites may be difficult to discern above the main effect of sex, as with our current analysis.

There are limitations of our work that should be addressed in future investigations, including a relatively small sample, fewer males than females, ethnic diversity (a confounder in DNAm data), and the absence of genetic variability. To account for these limitations, we applied corrections for multiple testing in order to avoid false positive results, focused on results that surpassed biological thresholds, controlled for ethnicity in all models, and checked available literature for possible effects of mQTLs. Another limitation is the use of self-reported Tanner stage, although these are moderately correlated with physicians’ physical examinations of pubertal development [28]. We note here that genetic association studies typically collapse analyses across the sexes, modeling sex as a covariate. Based on our results and on the existing literature on widespread sex differences in DNAm, future work should address the possibility of sex-specific mQTLs. One possible driver of the drastic sex differences across the autosomes is sex-specific genetic regulation.

## Conclusion

In this study, we explored sexual differentiation of the DNA methylome across the pubertal junction, with the goal of isolating and then comparing within-individual change over time and sex differences. We showed here that sex- related DNAm patterns join time-shifting sites in informing the biological processes of sexual dimorphism at puberty in humans. Targeting more subtle shifts in DNAm dynamics by examining correlated gene networks may help to uncover the hidden genetic architecture underlying sex differences in disease.

## Methods

Distributions of age and Tanner stage across time points for males and females are shown in Supplementary Fig. 1a, and distribution of testosterone in Supplementary Fig. 1b. The average time period between T1 and T2 was 1.97 years (sd = 0.33, min = 1.29, max 3.37). 54% of the sample identified as Caucasian, 6% as African American, 9% as Hispanic, 13% as Asian, 10% as biracial, and 8% as other.

Participants were recruited via media and online adverisements from the San Francisco Bay Area. Females were excluded if they had experienced the menses onset at T1. Pubertal stage was matched between males and females. Participants were additionally excluded if not fluent in English, as well as for MRI-related criteria, histories of neurological disordes or medical illnesses, or cognitive/physical limitations.

### Pubertal stage

Males and females were matched for pubertal stage at T1 using self-reported Tanner staging [42]. Self-report of Tanner staging is less invasive than physical examinations, and scores correlate with physical examinations of pubertal development by physicians [12, 43]. To report developmental stage, participants selected how closely their physical features resembled schematic drawings. Ratings were performed on a scale of 1 (prepubertal) to 5 (postpubertal). Averages of Tanner scores were used to index overall pubertal development as inclusion criteria at T1.

### Saliva samples

Participants provided saliva sample for DNAm quantification and gonadal hormone assays (Oragene Discover OGR-500). Upon confirming eligibility, participants were invited to return within one month to complete the fMRI portion of the study. Participants returned to the scan session with a saliva sample collected from passive drool upon awakening (while still lying in bed before breakfast or brushing teeth). Participants were re-contacted to return for their T2 session after ~ 24 months. All assessments conducted at T1 were repeated at T2.

### Gonadal hormones

Salivary hormonal assays were obtained for estradiol and progesterone (females) and DHEA and testosterone (all participants). Collected time was recorded by participants, and samples were placed their home freezer after collection. Participants returned the samples to be transferred to a − 20 °C freezer in the Psychology Department at Stanford University. Samples were shipped on dry ice to Salimetrics, LLC (State College, PA) for assays of salivary testosterone and estradiol (for details see [44]). As testosterone levels were not normally distributed, values were log-transformed for analysis. No female participants reported using hormonal contraceptives at T1 or T2.

### DNAm quality control

The EZ DNA Methylation Kit (Zymo Research, Irvine, CA, USA) bisulfite converted 750 ng of genomic DNA to differentiate unmethylated and methylated cytosine nucleotides based on sequence. A total of ~ 160 ng of bisulfite-converted DNA was whole-genome amplified and enzyme fragmented, and then hybridized to a MethylationEPIC BeadChip (‘EPIC array’; Illumina, San Diego, CA, USA). The EPIC array is a genome-wide platform assessing ~ 850,000 CpG sites. PBeadChips were scanned on an Illumina HiScan. Initial quality assessment was performed in GenomeStudio (Illumina) on intensity values as well as color correction and background subtraction. A data matrix of beta values was exported representing percent DNAm (0 to 1; 0 = unmethylated and 1 = methylated). Next, a probe filtering step removed SNP probes, polymorphic probes, cross hybridizing probes [45], and probes with missing values or detection *p* values >1e^16^. After filtering, 794,811 probes remained within the autosomes, 17,4660 in the X chromosome, and 31 in the Y chromosome. Probes on the X and Y chromosomes for males, and X chromosomes for females were separated from the autosomes for the remainder of preprocessing and data analysis. Next, because the Illumina platform includes two probe types (Type I and II) with different distributions, data normalization was performed using beta mixture quantile method [46].

The cellular content of saliva samples was estimated and compared between two reference-based cell-type deconvolution methods, including Hierarchical Epigenetic Dissection of Intra-Sample-Heterogeneity (HepiDISH) [47] and an earlier deconvolution algorithm [48]. Because HepiDISH estimated variance in fibroblasts, and correlations were extremely high between HepiDISH estimates of epithelial cells and proportions of epithelial cells and CD34 cells estimated by Smith’s method, we carried forward with estimates of fibroblasts and epithelial cells from HepiDISH, which were not correlated with one another and captured substantial proportions of variance (Supplementary Fig. 4), as expected. Predicted epithelial cell proportions were significant related to time point (standardized β = 0.34, *p* = 0.01), but proportions were accounted for in all analyses.

Finally, beta values were batch corrected for the effects of chip using ComBat from the SVA package [49]. To inform covariates in statistical models, quality controlled beta values from T1 and T2 were visualized by performing principal component analysis and plotting the top PCs against variables, shown in Supplementary Fig. 4.

### Statistical analyses

Statistical models were run in ‘limma,’ linear models for microarray data [50] and mixed models used the lme4 package. Ethnicity was coded as a factor variable, with all categories included. Sex models controlled for age, ethnicity and cell type. Smoking was only endorsed by two participants at time 2, and post hoc checks confirmed that final significant sex sites meeting biological thresholds were not changed by smoking status. Moreover, no sites from time or sex models carried forward for network analysis were located within genes implicated in smoking effects on DNAm in prior work (i.e., *AHRR*, *RARA*, *F2RL3*, and *LRRN3;* [51]*).* Mixed time models tested for the effect of timepoint, controlling for the interval in years between time points, ethnicity and cell type, with individual added as a random effect. Cell types were checked for interindividual correlation between time point 1 and time point 2 and found to be nonsignificant (Fibroblasts r = .07, *p* = .43; Epithelial cells: r = .18, *p* = .05). Follow up mixed models further assessed the interaction between age gap and time at all DNAm sites tested (794,811), and found one significant site after multiple test correction. This suggests that overall, the effect of timepoint were not dependent on the interval of time between DNAm measurements. To follow up on the significant sites meeting biological thresholds from these mixed models testing the effect of time, we used delta or difference values between time 1 and 2, and prediction by the time interval (in years), sex, and Tanner stage change. These models used principal components of cell type proportions measured at time 1 and time 2 to ensure that individual-level correlations between cell type proportions at the two time points were accounted for. DNAm change at a total of 20 sites were significantly predicted by age interval, showing that some DNAm changes identified by the time models did correspond with changes in age. One site was predicted by sex, and no sites were predicted by change in Tanner stage. All R code for data analysis, results, and Figure production is available on the corresponding author’s GitHub account (srm254).

### Gene ontology functional pathway analysis

The Bioconductor package “missMethyl” [52] was used to explore the gene ontologies of sets of probes over represented relative to all probes explored (794,811 CpGs or the ‘Cpg universe’). Significance thresholds were set to *p* < 0.001 to obtain the top associated biological processes.

### Weighted correlation network analysis (WGCNA)

WGCNA reduces patterns of co-methylated loci by estimating gene clusters or ‘modules’ [53] using hierarchical clustering trees. Modules are summarized by their first principal component or eigengenes, which represent how modules relate to one another across samples. We explored the overlap of the 10 most highly connected probes in each module, identified as “hubs” and for pathway analysis, we also incorporated the most highly connected CpGs using a KME (eigen-based connectivity) thresholds of 0.7.

### Enrichments for genomic context, mQTLs, and androgen response elements

For testing enrichment for genomic location, each quality controlled CpG site with more than one transcript was annotated to the largest associated transcript. CpG sites related to sex (723 sites) and time (566 sites) were compare to the full background of tested sites (794,811 sites) on the EPIC array for enrichment for genomic locations. A two-proportion Z-test was used to compare the proportion of sex or time DNAm sites associated with a given location relative to the proportion from all quality controlled probes.

We compared our results for overlap with previously reported methylation quantitative trait loci (mQTLs) in buccal cells [54], which contain a similar composition of epithelial cells as saliva [55]. Summary statistics were obtained from the corresponding author of a prior publication [54] to test for overlap of sex- and time-related CpG sites with previously reported methylation quantitative trait loci (mQTLs). The *GeneOverlap*^55^ package in R was used to obtain odds ratios and *p* values from the Fisher’s exact test for overlap relative to quality controlled EPIC array probes (794811). We assessed the overlap of our significant sites that surpassed biological threshold with significant mQTLs within a distance of 1 Mb in a recent study of buccal cells in a sample of males and females (mean age 7.5 years) [54]. Out of 723 sex-related sites, 67 (9.27%) were associated with an mQTL in buccal cells, which is more than would be expected by chance (OR = 2.3, overlapping *p* value = 4.9e^− 09^), and 45 out of 566 time-related sites were associated with mQTLs (7.96%; OR = 1.9, overlapping p value = 5.6e^− 05^). These results suggest that genetic variation plays a role in both sex differences and time-related shifts in DNAm.

The coordinates for androgen response elements were downloaded from remap and a two-proportion Z-test was used to compare the proportion of sex (85%) or time (87%) DNAm sites that were found within androgen response elements to the proportion of randomly selected DNAm sites from quality controlled probes (average from 10 random draws of 723 probes = 335.7 probes; 46%).

## Supplementary information

**Additional file 1: Fig. S1.** Distributions of age, tanner stage, and testosterone in males and females.

**Additional file 2: Fig. S2.** A) Correlations between sex DNAm modules (‘blue’ and ‘turquoise’) composed of all *p* value significant probes and DNAm modules composed of all biologically thresholded probes. B) Correlations between time DNAm modules (‘magenta’, ‘yellow’, and ‘brown’; note ‘grey’ represent unassigned sites that do not nest together in a module) composed of all p value significant probes and modules composed of all biologically thresholded probes.

**Additional file 3: Fig. S3.** Correlations of hub probes for blue and turquoise modules from WGCNA analysis of sex-related probes with testosterone at T1 and T2. Correlations with testosterone become stronger at T2 (right) relative to T1 (left). *Note*. Blue module on top; Turquoiose module on the bottom.

**Additional file 4: Fig. S4.** Visualization of correlations between 1) biological variables, technical batch variables, and covariates and 2) DNAm principal components summarizing the variability of all autosomal beta values after preprocessing. The lack of correlations with Sentrix ID (chip) and Sentrix Position (row) confirms effective technical batch correction. ‘Epi’ = epithelial cells and ‘Fib’ = fibroblasts estimated by HepiDISH.

**Additional file 5: Table S1.** All significant model results and relevant annotation information for the effect of 1) sex at T1, 2) sex at T2, 3) time effects 4) follow up models of time-related sites, 5) testosterone effects. **Table S2.** Genomic context enrichment results. **Table S3.** Details on kMeans threshold probes for modules identified in the 1) sex analysis and 2) time analysis. **Table S4.** Gene Ontology enrichment analyses results for probes belonging to gene modules based on kMeans threshold. **Table S5.** Interaction model results.

## Data Availability

The datasets generated and analysed during the current study are available in the GEO data repository, GEO accession: GSE150643 available at https://www.ncbi.nlm.nih.gov/geo/query/acc.cgi?acc=GSE150643. The remap data base provides coordinates for androgen response elements: http://pedagogix-tagc.univ-mrs.fr/remap/factor.php?TF=AR&page=overview. Methylation quantitative trait loci were compared to a previously published data set: https://epigeneticsandchromatin.biomedcentral.com/articles/10.1186/s13072-018-0225-x provided by contacting the corresponding author.

## Abbreviations

DNAm: DNA methylation
CpG: Cytosine base adjacent to a guanine base
T1: Time 1
T2: Time 2
WGCNA: Weighted correlation network analysis
