# Supplementary figures and images for: Distinctions between sex and time in patterns of DNA methylation across puberty
Source: BMC Genomics. 2020 Jun 3;21:389. doi: 10.1186/s12864-020-06789-3 (PMC7268482; doi:10.1186/s12864-020-06789-3)

**Age Time 1**

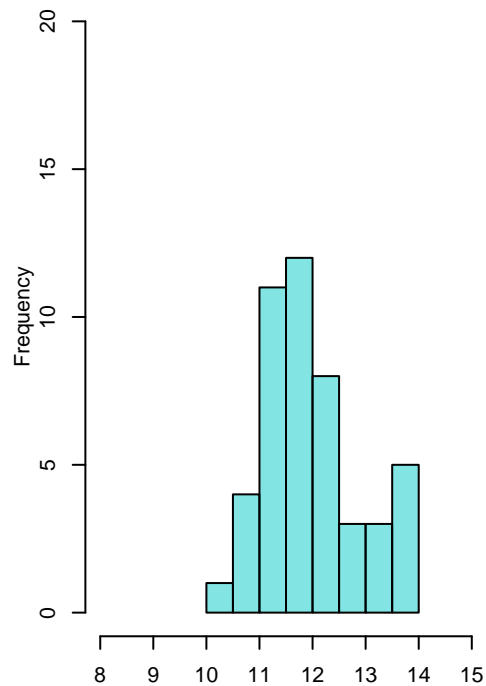

**Age Time 2**

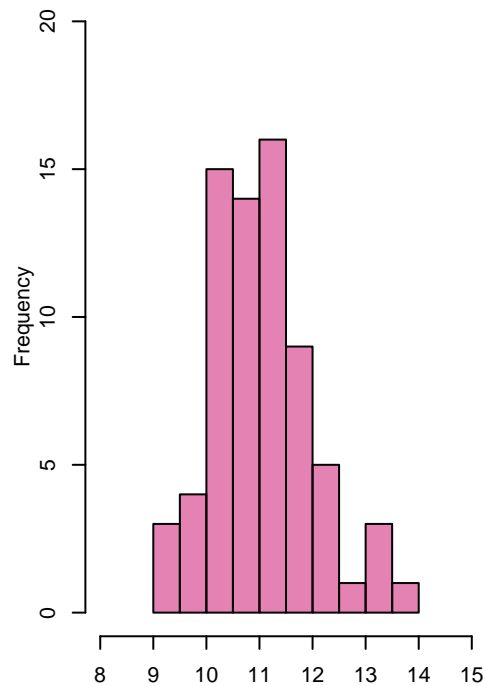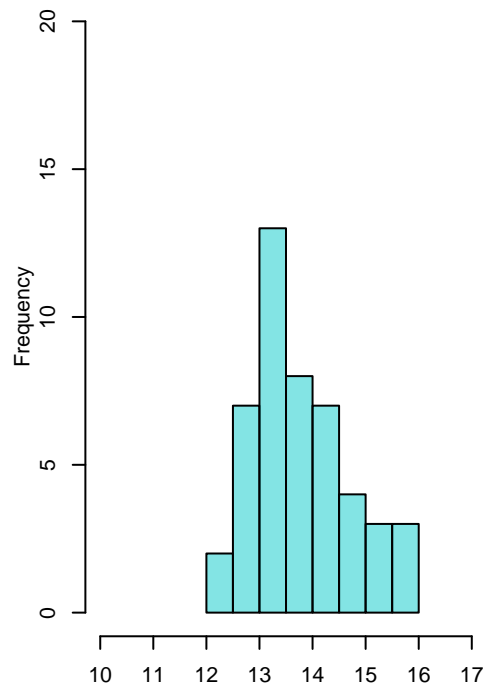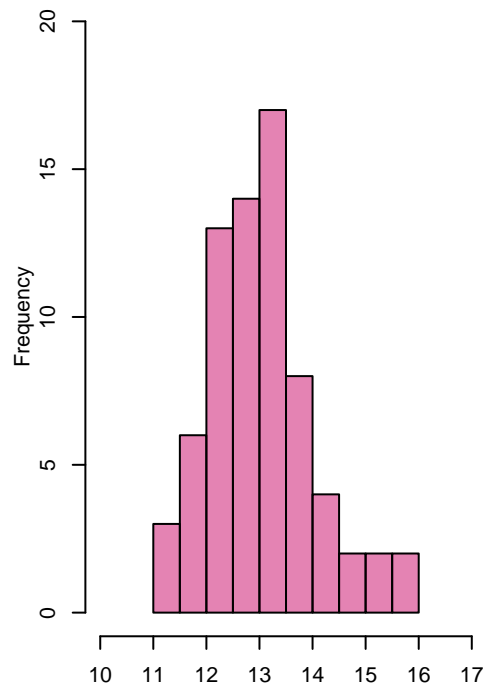

**Tanner Stage Time 1**

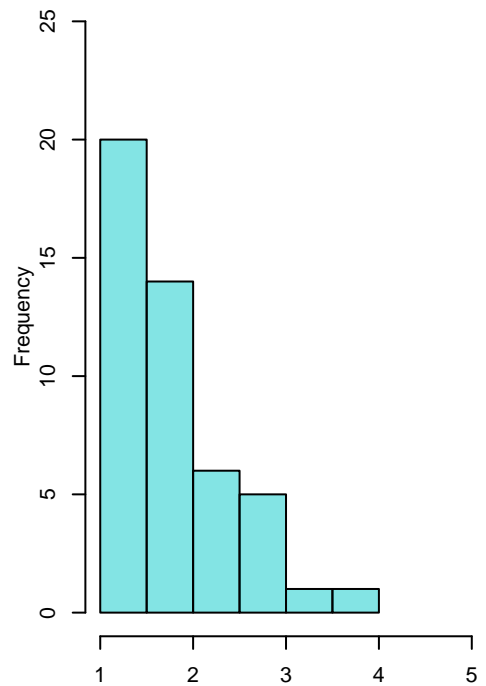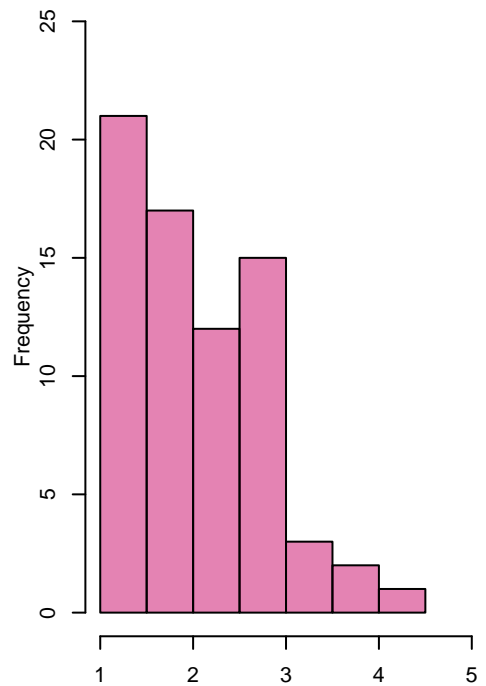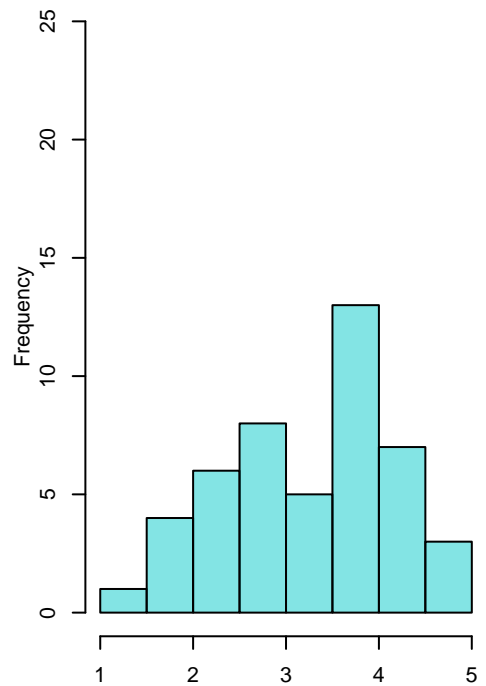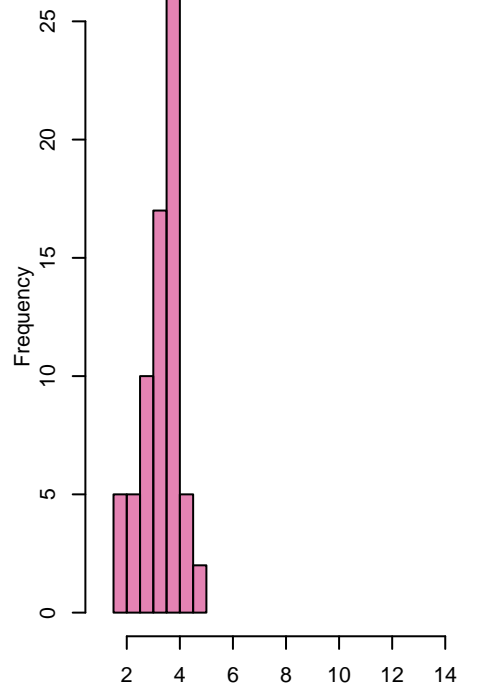

Supplement: Supplementary file 1 — Additional file 1: Fig. S1. Distributions of age, tanner stage, and testosterone in males and females. [file 12864_2020_6789_MOESM1_ESM.zip › supp1a_figure_separated.pdf]

**Testosterone Time 1**

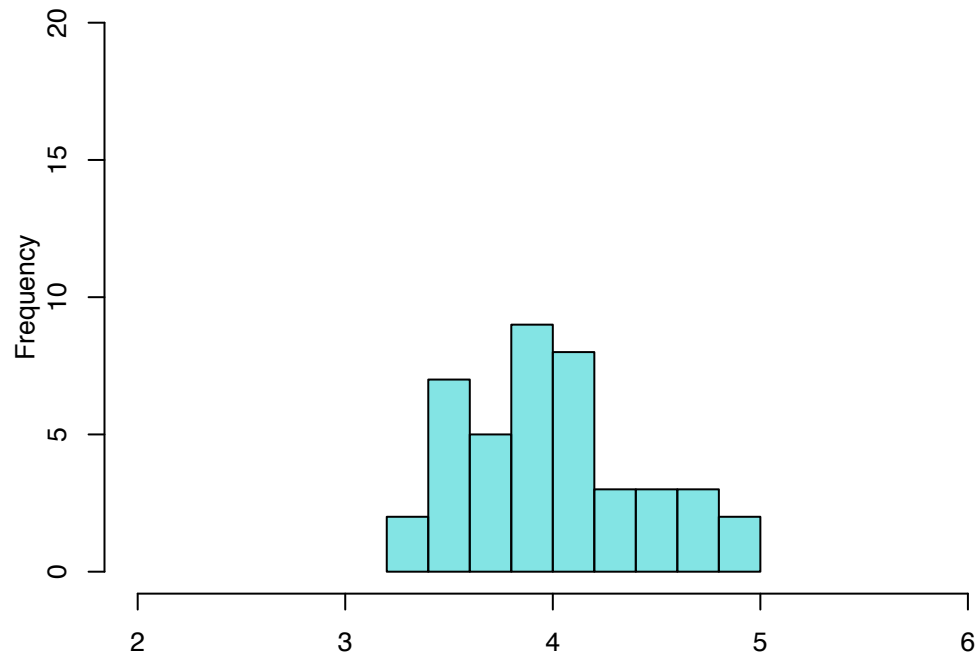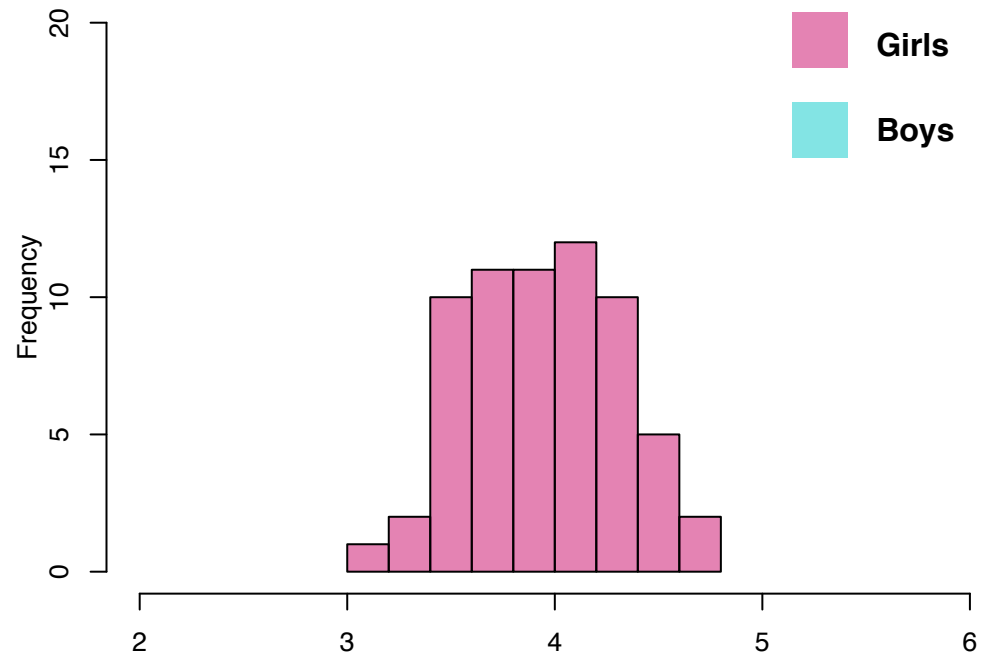

**Testosterone Time 2**

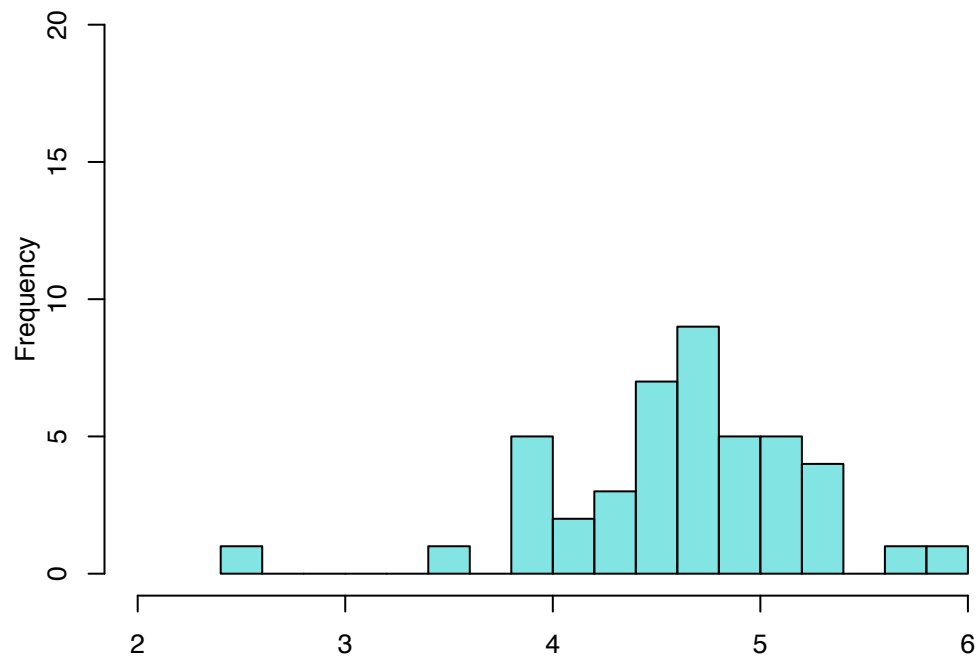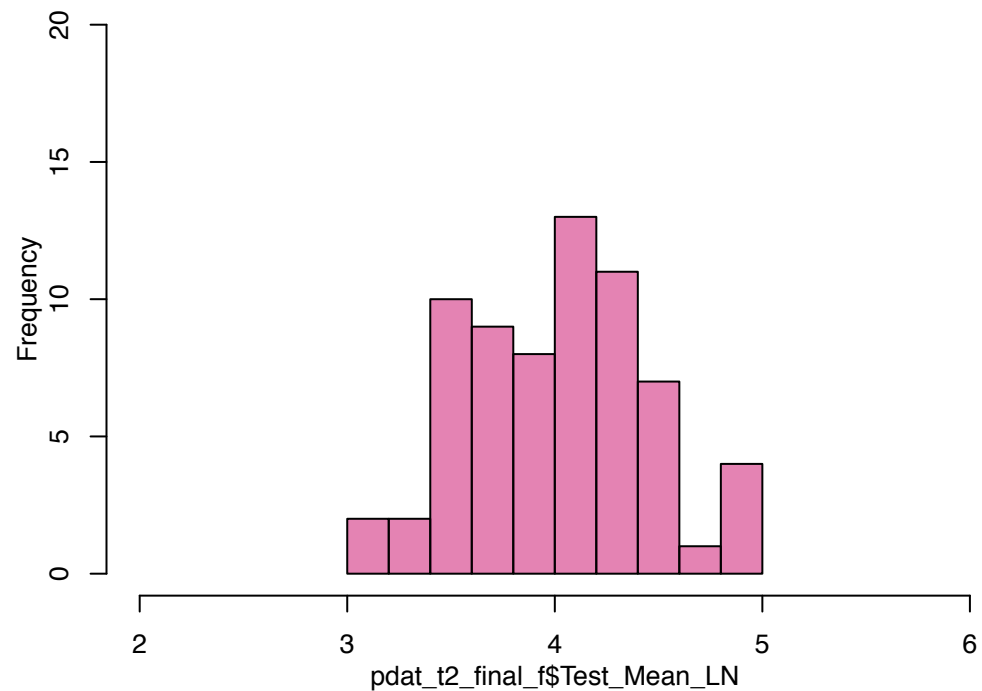

Supplement: Supplementary file 1 — Additional file 1: Fig. S1. Distributions of age, tanner stage, and testosterone in males and females. [file 12864_2020_6789_MOESM1_ESM.zip › Supp1btestosterone_histogram.pdf]

**A**

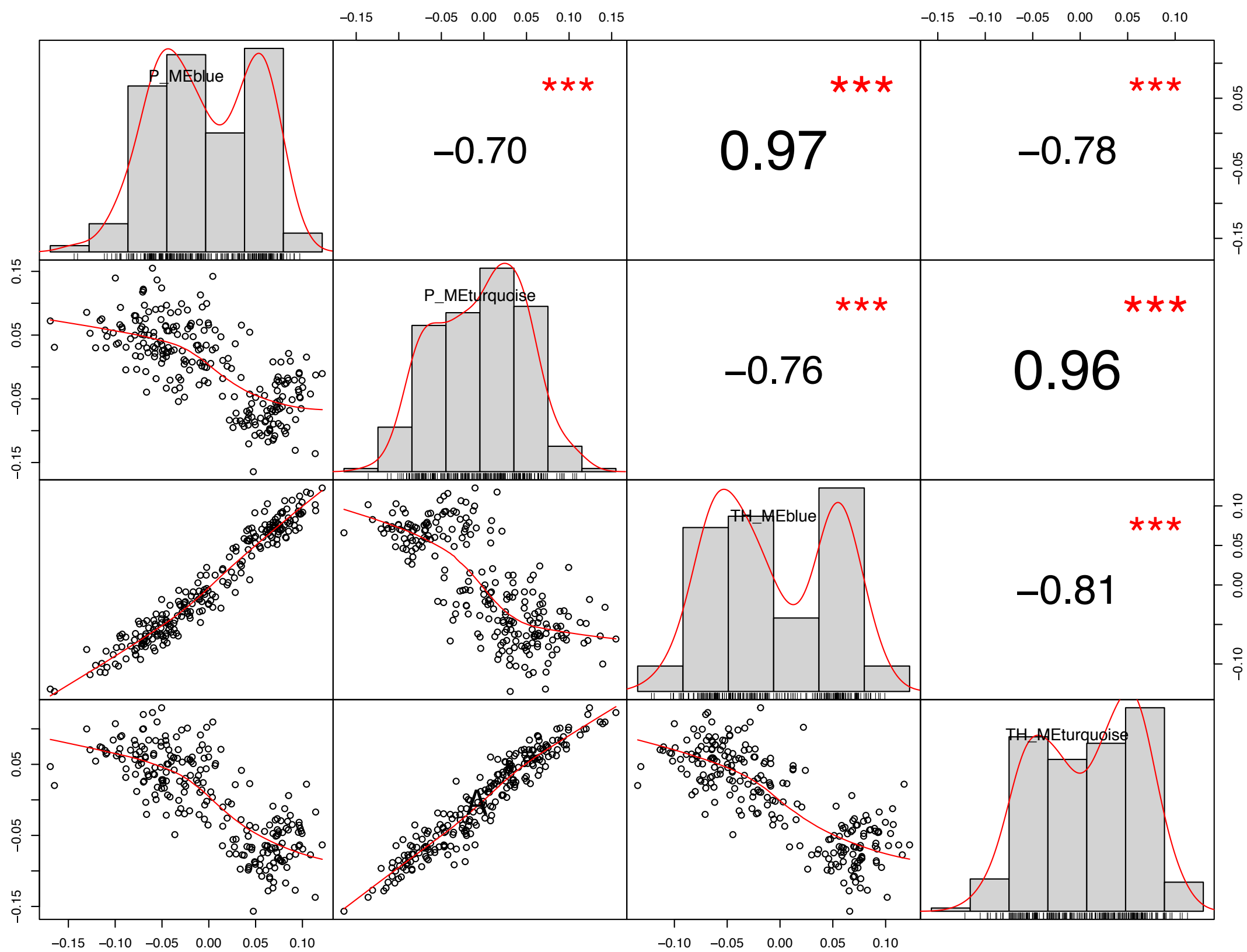

# B

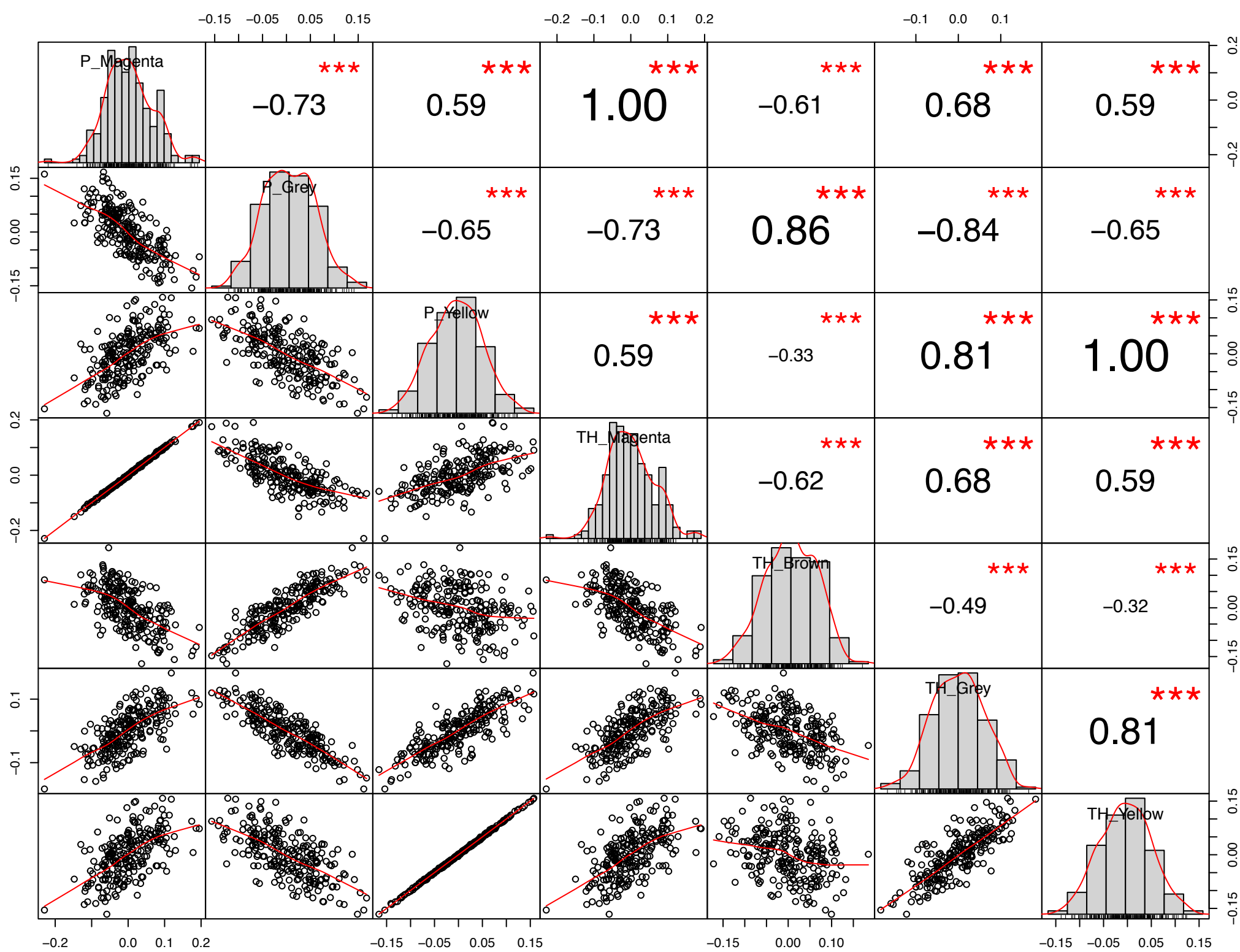

Supplement: Supplementary file 2 — Additional file 2: Fig. S2. A) Correlations between sex DNAm modules (‘blue’ and ‘turquoise’) composed of all p value significant probes and DNAm modules composed of all biologically thresholded probes. B) Correlations between time DNAm modules (‘magenta’, ‘yellow’, and ‘brown’; note ‘grey’ represent unassigned sites that do not nest together in a module) composed of all p value significant probes and modules composed of all biologically thresholded probes. [file 12864_2020_6789_MOESM2_ESM.pdf]

A

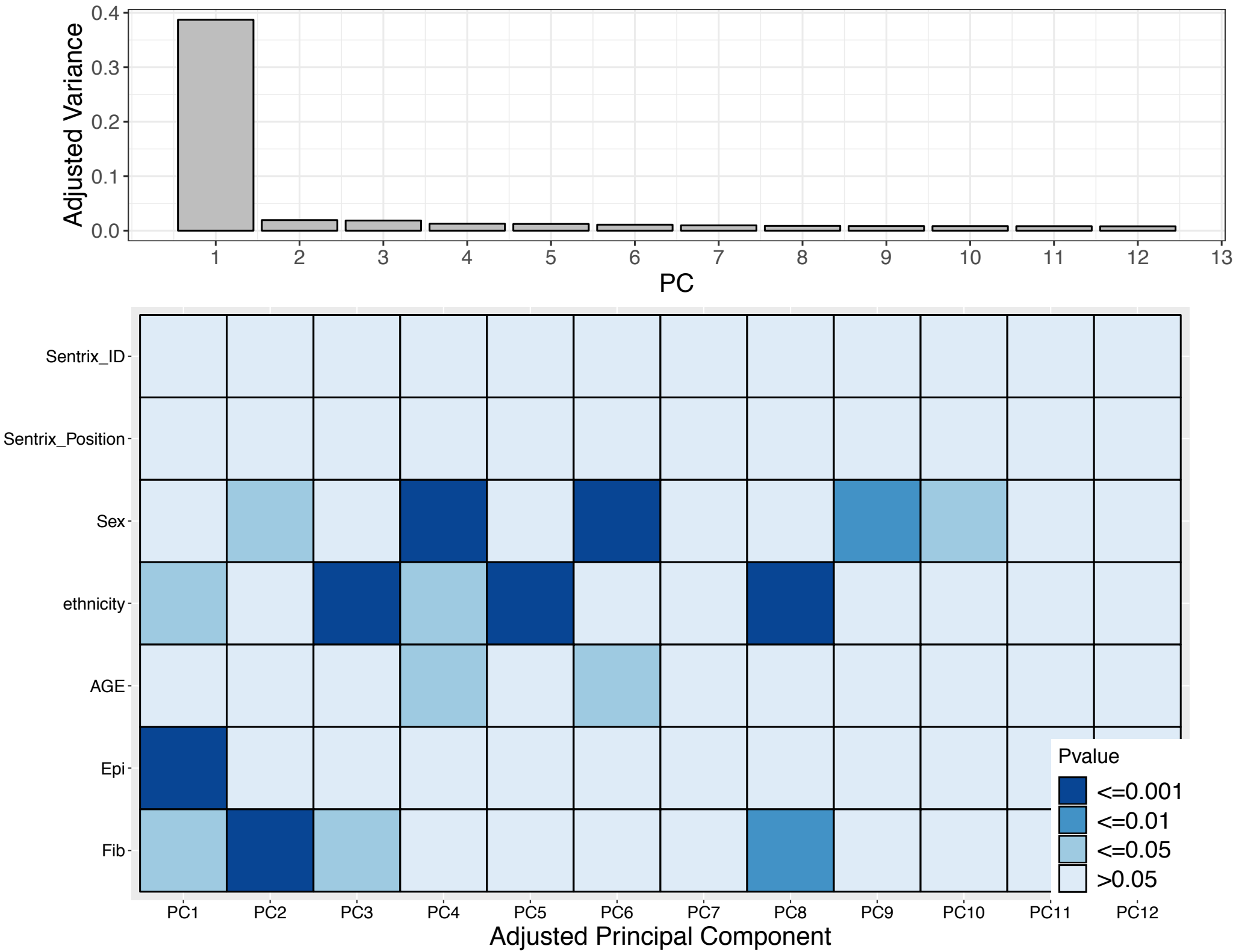

B

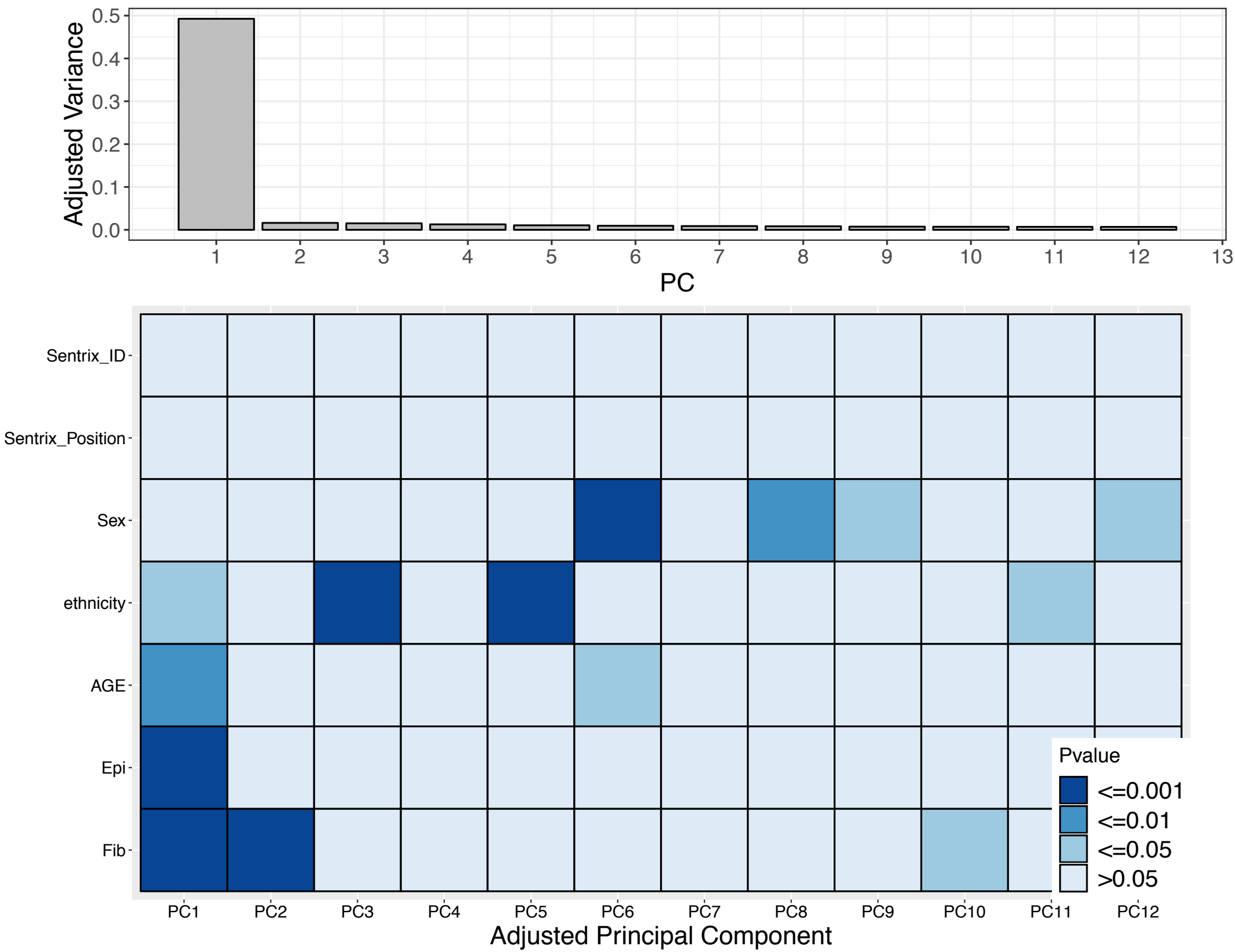

Supplement: Supplementary file 4 — Additional file 4: Fig. S4. Visualization of correlations between 1) biological variables, technical batch variables, and covariates and 2) DNAm principal components summarizing the variability of all autosomal beta values after preprocessing. The lack of correlations with Sentrix ID (chip) and Sentrix Position (row) confirms effective technical batch correction. ‘Epi’ = epithelial cells and ‘Fib’ = fibroblasts estimated by HepiDISH. [file 12864_2020_6789_MOESM4_ESM.pdf]
