# Supplementary material for: Distinctions between sex and time in patterns of DNA methylation across puberty
Source: BMC Genomics. 2020 Jun 3;21:389. doi: 10.1186/s12864-020-06789-3 (PMC7268482; doi:10.1186/s12864-020-06789-3)
Supplement: Supplementary file 3 — Additional file 3: Fig. S3. Correlations of hub probes for blue and turquoise modules from WGCNA analysis of sex-related probes with testosterone at T1 and T2. Correlations with testosterone become stronger at T2 (right) relative to T1 (left). Note. Blue module on top; Turquoiose module on the bottom. [file 12864_2020_6789_MOESM3_ESM.pdf]

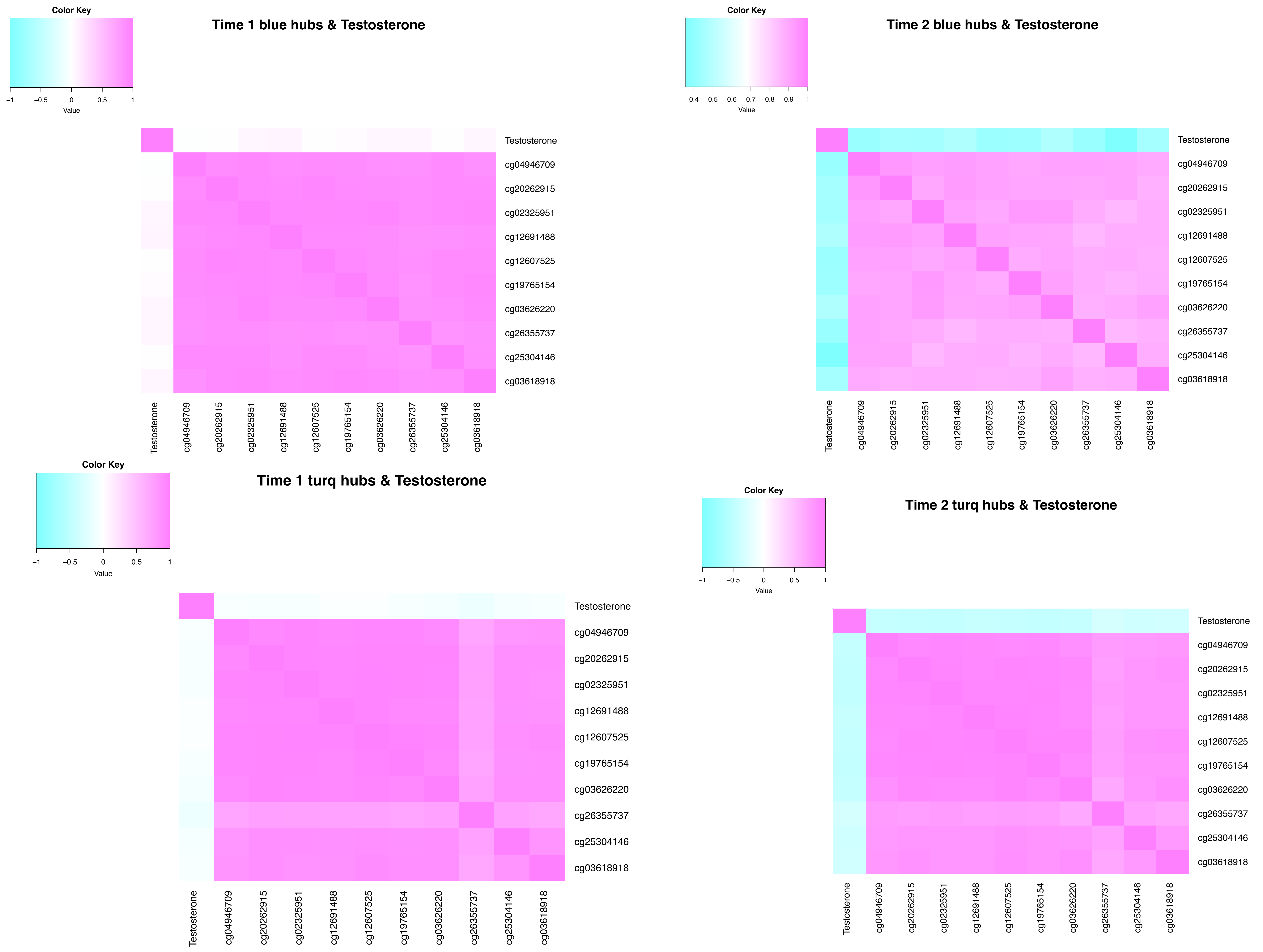
Time 1 blue hubs & Testosterone
Color Key
−1 −0.5 0 0.5 1
Value
Testosterone
cg04946709
cg20262915
cg02325951
cg12691488
cg12607525
cg19765154
cg03626220
cg26355737
cg25304146
cg03618918
Time 2 blue hubs & Testosterone
Color Key
0.4 0.5 0.6 0.7 0.8 0.9 1
Value
Testosterone
cg04946709
cg20262915
cg02325951
cg12691488
cg12607525
cg19765154
cg03626220
cg26355737
cg25304146
cg03618918
Time 1 turq hubs & Testosterone
Color Key
−1 −0.5 0 0.5 1
Value
Testosterone
cg04946709
cg20262915
cg02325951
cg12691488
cg12607525
cg19765154
cg03626220
cg26355737
cg25304146
cg03618918
Time 2 turq hubs & Testosterone
Color Key
−1 −0.5 0 0.5 1
Value
Testosterone
cg04946709
cg20262915
cg02325951
cg12691488
cg12607525
cg19765154
cg03626220
cg26355737
cg25304146
cg03618918
